# Anticorrosive and Microbial Inhibition Performance of a Coating Loaded with *Andrographis paniculata* on Stainless Steel in Seawater

**DOI:** 10.3390/molecules26113379

**Published:** 2021-06-03

**Authors:** Wan Mohamad Ikhmal Wan Mohamad Kamaruzzaman, Maria Fazira Mohd Fekeri, Nursabrina Amirah Mohd Nasir, Nur Aiman Syafiq Mohd Hamidi, Mohamad Zahid Baharom, Azila Adnan, Muhamad Syaizwadi Shaifudin, Wan Rafizah Wan Abdullah, Wan Mohd Norsani Wan Nik, Fariza Hanim Suhailin, Khamirul Amin Matori, Chen Soo Kien, Mohd Hafiz Mohd Zaid, Mohd Sabri Mohd Ghazali

**Affiliations:** 1 Faculty of Science and Marine Environment, Universiti Malaysia Terengganu, Kuala Nerus 21030, Terengganu, Malaysia; ikhmal007@gmail.com (W.M.I.W.M.K.); mariafazirafekeri@gmail.com (M.F.M.F.); sabrinaamirah38@gmail.com (N.A.M.N.); aimansyafiq140@gmail.com (N.A.S.M.H.); mohdzahidbaharom@gmail.com (M.Z.B.); azila.adnan@umt.edu.my (A.A.); syaizwadi@gmail.com (M.S.S.); 2 Advanced Nano Materials (ANoMa) Research Group, Faculty of Science and Marine Environment, Universiti Malaysia Terengganu, Kuala Nerus 21030, Terengganu, Malaysia; farizahanim@utm.my; 3 Materials Synthesis and Characterization Laboratory (MSCL), Institute of Advanced Technology, Universiti Putra Malaysia, Serdang 43400, Selangor, Malaysia; wanrafizah@umt.edu.my (W.R.W.A.); chensk@upm.edu.my (C.S.K.); 4 Faculty of Ocean Engineering Technology and Informatics, Universiti Malaysia Terengganu, Kuala Nerus 21030, Terengganu, Malaysia; niksani@umt.edu.my; 5 Physics Department, Faculty of Science, Universiti Teknologi Malaysia (UTM), Skudai 81310, Johor Bahru, Malaysia; 6 Department of Physics, Faculty of Science, Universiti Putra Malaysia, Serdang 43400, Selangor, Malaysia; khamirul@upm.edu.my (K.A.M.); mhmzaid@upm.edu.my (M.H.M.Z.)

**Keywords:** paint coatings, microbiological corrosion, *Andrographis paniculata*, stainless steel, seawater

## Abstract

With the trend for green technology, the study focused on utilizing a forgotten herb to produce an eco-friendly coating. *Andrographis paniculata* or the kalmegh leaves extract (KLE) has been investigated for its abilities in retarding the corrosion process due to its excellent anti-oxidative and antimicrobial properties. Here, KLE was employed as a novel additive in coatings and formulations were made by varying its wt%: 0, 3, 6, 9, and 12. These were applied to stainless steel 316L immersed in seawater for up to 50 days. The samples were characterized and analyzed to measure effectiveness of inhibition of corrosion and microbial growth. The best concentration was revealed to be 6 wt% KLE; it exhibited the highest performance in improving the ionic resistance of the coating and reducing the growth of bacteria.

## 1. Introduction

Corrosion is a natural phenomenon that involves oxidation and reduction in the presence of water and oxygen and leads to the degradation and deterioration of the metal [1]. Several chemical, physical, electrochemical, and microbiological factors influence the corrosion rate, such as the redox reaction, temperature or velocity of the medium, the electrode potential of the substrate, and formation of the biofilm by micro- and macroorganisms, respectively [2]. In the marine industry, problems including contamination and the breakdown of pipelines caused by the thinning of the innermost layer due to corrosion are common. The breakdown of a pipeline not only results in a higher cost of maintenance and repair, but may also cause pollution due to oil spillage [3]. To address this problem, the use of a coating that protects materials from interacting with the environment is essential [4]. Additionally, corrosion in seawater can be accelerated by the interaction of the metal with living organisms such as the sulfate-reducing bacteria and by specific seawater parameters such as the salinity. The salinity, particularly chloride ions, of seawater is considered a contributing factor in the degradation of coatings [5]. The degradation or failure of a coating begins when electrolytes penetrate the coating layer by forming small holes that allow electrolytes to enter and make contact with the metal, causing the corrosion reaction to take place [6].

Seawater also contains microorganisms known as corrosion-relevant microorganisms, mainly bacteria that promote the biocorrosion process at a rapid rate. In an aqueous environment, microorganisms are broadly distributed over every layer of the ocean. In such conditions, immersion of a sample at any depth induces bacteria to attach to the surface of the metal and form a slimy layer of the biofilm, which is the main cause of the deterioration and degradation of the material [7,8]. A few types of bacteria induce microbiologically influenced corrosion (MIC), such as sulfate-reducing bacteria (SRB) and iron-reducing bacteria (IRB). The SRB typically represent the anaerobic type of bacteria, while the IRB represent the aerobic type. In most cases, SRB are considered to be more harmful since no other groups of bacteria produce a comparable corrosion damage when evaluated in laboratory-grown pure cultures [9]. In this study, two types of bacteria have been chosen for the investigation of the antimicrobial properties of the coating: *Staphylococcus aureus* (+), Gram-positive bacteria, and *Pseudomonas aeruginosa* (−), Gram-negative bacteria. *S. aureus* (+) is commonly found on land, but it can also be found in seawater. *S. aureus* (+) has also been reported to influence corrosion over the course of time [10]. Similarly, *P. aeruginosa* (−) has been observed to cause severe corrosion damage to carbon steel in a seawater medium [11].

This study aims to develop a new coating with a plant extract as an additive that enhances its anticorrosion and antimicrobial properties. Several studies have used plant extracts as additives, such as cauliflower [12,13] and *Leucaena leucocephala* [14]. Moreover, the use of natural products from the flora and fauna is believed to be non-harmful, non-toxic, biodegradable, and readily available for mass production [15]. The bioactive compounds in the plants, such as alkaloids, flavonoids, and gallic acids, are believed to retard the corrosion process. Plants are mostly antioxidation agents rich in phenolic compounds that can help slow down the corrosion process by retarding the oxidation reaction. Furthermore, plant extracts can be easily mixed into the coating system and produce strong barriers to protect metal surfaces [16,17]. In particular, the extract of *Andrographis paniculata* has been employed as an additive, along with other components. *A. paniculata* is also known as kalmegh in India. Commonly, it is called as the ‘king of bitters’ due to its strong bitter taste. This herbaceous plant is mostly found in subtropical Asia, Southeast Asia, and India [18,19]. *A. paniculata* has been known to have antioxidation and antimicrobial properties [20,21]. The combination of the *A. paniculata* leaves extract with zinc oxide and calcium carbonate as pigments, methyl isobutyl ketone as a solvent, and WW rosin as a binder is believed to ensure great performance in inhibiting corrosion and microbial progress on stainless steel grade 316L (SS316L) so as to increase its service life period.

## 2. Results and Discussion

### 2.1. Optical Studies

#### 2.1.1. Fourier-Transform Infrared (FTIR) Spectroscopy

**Figure 1:**
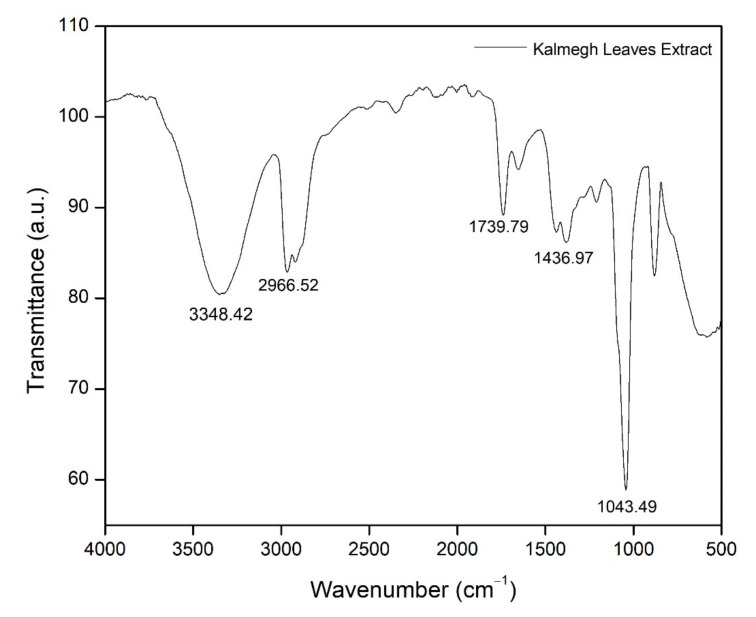
FTIR spectrum of the KLE.

**Figure 2:**
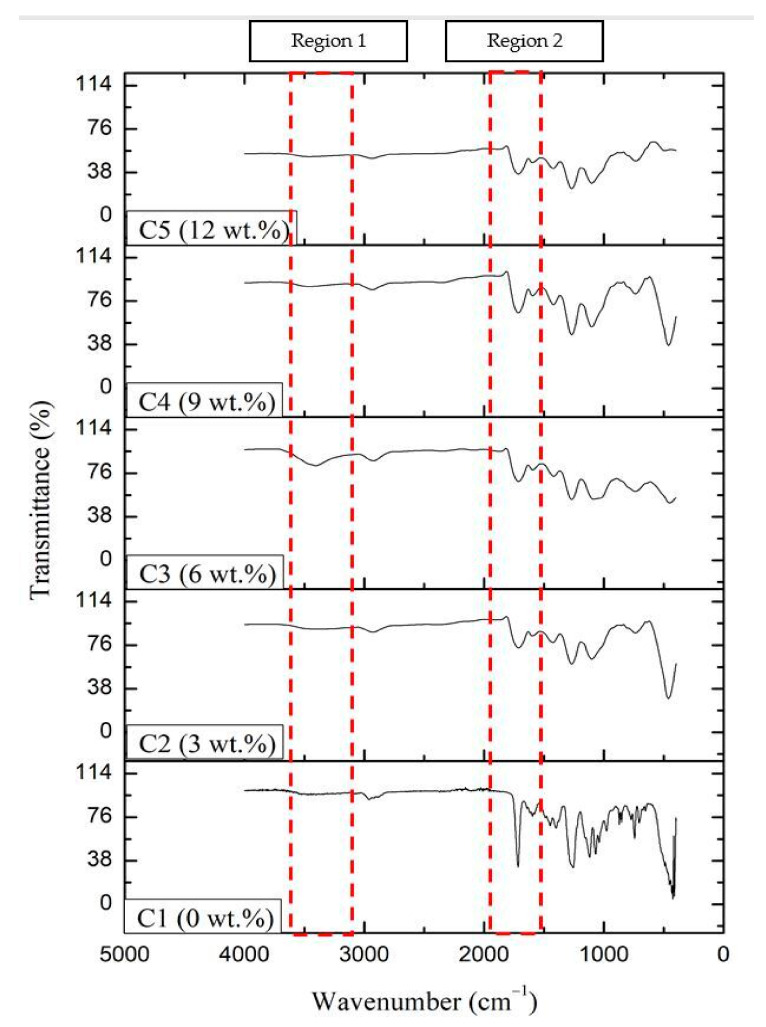
FTIR spectra of coatings with different wt% of the KLE.

According to Figure 1 and Table 1, the functional groups observed based on the obtained FTIR spectrum of the KLE include primary alcohol, carboxylic acid, carbonyl, alkane, and ethers. At wavenumber 3348.42 cm^−1^, a strong and broad peak was observed, representing the alcohol or phenolic group (O−H) stretching vibration. A strong peak from the carboxylic acid (R−COOH) was observed at wavelength 2966.52 cm^−1^. The amide (C=O) group was represented at wavenumber 1739.79 cm^−1^. At 1436.97 cm^−1^, the peak present is that of alkane (C−H), whereas at 1043.49 cm^−1^, vibration of the ether’s group (R−O−R) was detected. The functional groups, namely O−H, C=O, C−H, and R−COOH, have similar characteristics toward andrographolide, the major compound in the KLE [22,23,24]. Figure 2 shows the FTIR spectrum of five different coatings labeled C1, C2, C3, C4, and C5. Based on region 1 (3650 cm^−1^ to 3253 cm^−1^), the intensity of the O−H peak was only significantly visible on specimen C3 compared to the rest of the specimens. This indicates that at C3, O−H peaks appear to be broad, resulting from the good mixing between the KLE and the coating matrix. In region 2 (1905 cm^−1^ to 1598 cm^−1^), the C=O group is present. Its intensity appears reduced because of the addition of the KLE in the coating matrix, as seen in the difference of sharpness between C1 and other coatings. The C=O intensity of C2 until C5 appears with no significant changes. These results suggest that the presence of the KLE does not dissociate nor diminish the C=O group in the coating matrix. A similar study has shown that the presence of C=O and O−H can lead to the formation of hydrogen bonds [25].

#### 2.1.2. X-Ray Diffraction (XRD)

**Figure 3:**
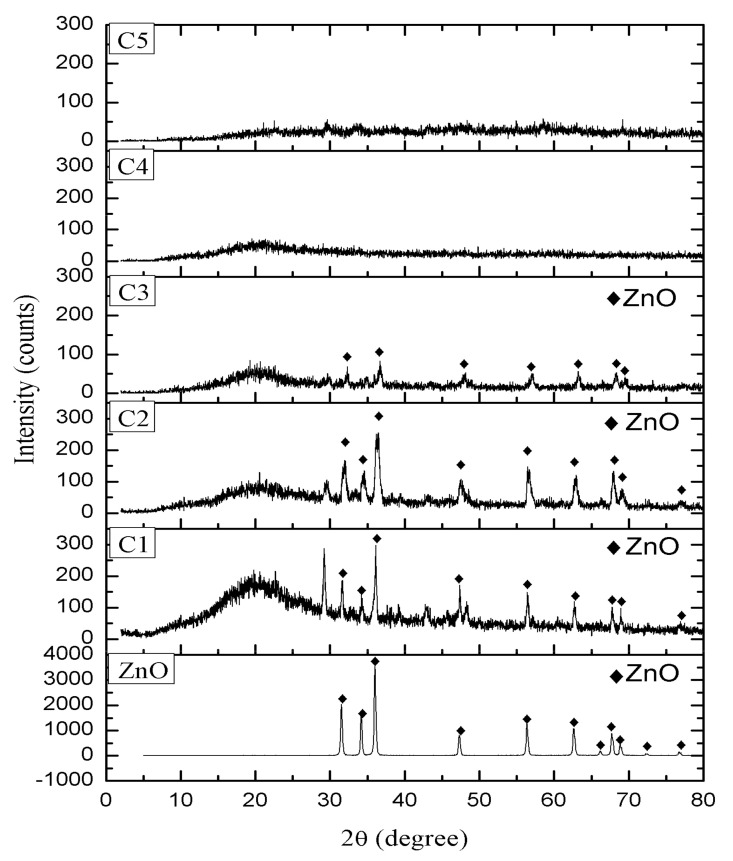
XRD diffraction of ZnO and the coatings.

The presence of zinc oxide (ZnO) and addition of the KLE were studied using XRD. Figure 3 shows the XRD of ZnO, displaying a few intensities at (100), (002), (101), (102), (110), (103), (200), (112), (201), (004), and (202) with the angle of 2θ at 31.11°, 33.79°, 35.73°, 47.05°, 56.15°, 62.41°, 65.84°, 67.48°, 68.68°, 72.11°, and 76.44°, according to PDF No. 80−74. ZnO is a crystalline material in the coating formulation, and its presence should increase stability of the coating’s performance [26]. Figure 3 shows the XRD diffractogram of ZnO and the coatings with and without KLE additives. A hump appeared between 20° and 30° at the low position of 2θ. The amorphous peak was possibly caused by abundance of the polymer (WW rosin) in the coating [27]. The diffraction pattern of ZnO appeared in the coatings with 0 wt%, 3 wt%, and 6 wt% of the KLE but diminished as the additive concentrations increased to 9 wt% and 12 wt%. The trend was proportional to the decreasing concentration of the ZnO powder with the increase in the concentration of the KLE. The addition of more than 6 wt% KLE and less than 14 wt% ZnO leads to a dispersal and reduction of the crystallinity of the coating. This confirms a previous study’s findings that a plant extract in the coating matrix has a limited upper value. When an amount greater than this upper limit is added, the performance of the paint starts to decrease, thus affecting its crystalline structure [13].

#### 2.1.3. High-Performance Liquid Chromatography

**Figure 4:**
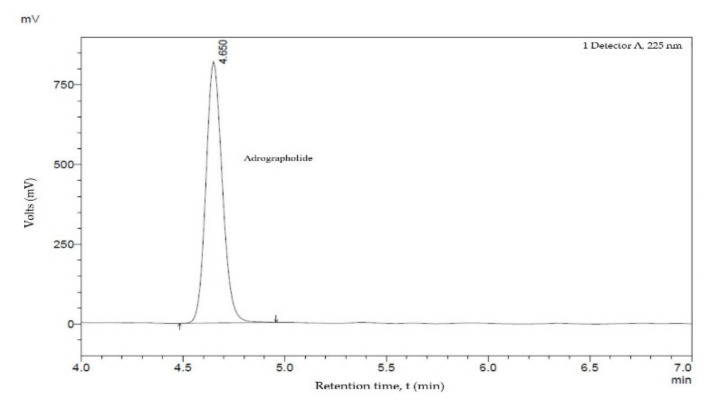
Chromatogram of the andrographolide compound.

In the current study, the diluted sample of the KLE was analyzed using HPLC to identify its major compound, andrographolide. The sample was observed using the mobile phase of water (20%) and acetonitrile (80%), where a retention time of 4.65 min was identified. The study was conducted by employing a wavelength of 225 nm with the peak area for the analyzed compound of 4,589,710 mV, as shown in Figure 4 and Table 2. The retention time of the identified andrographolide (C_20_H_30_O_5_) was compared with the standard based on a previous study to prove its validity [28]. Additionally, the concentration was considered unknown for the study since no external standard required to plot the calibration curve was available during the characterization.

### 2.2. Electrochemical Impedance Spectroscopy (EIS)

**Figure 5:**
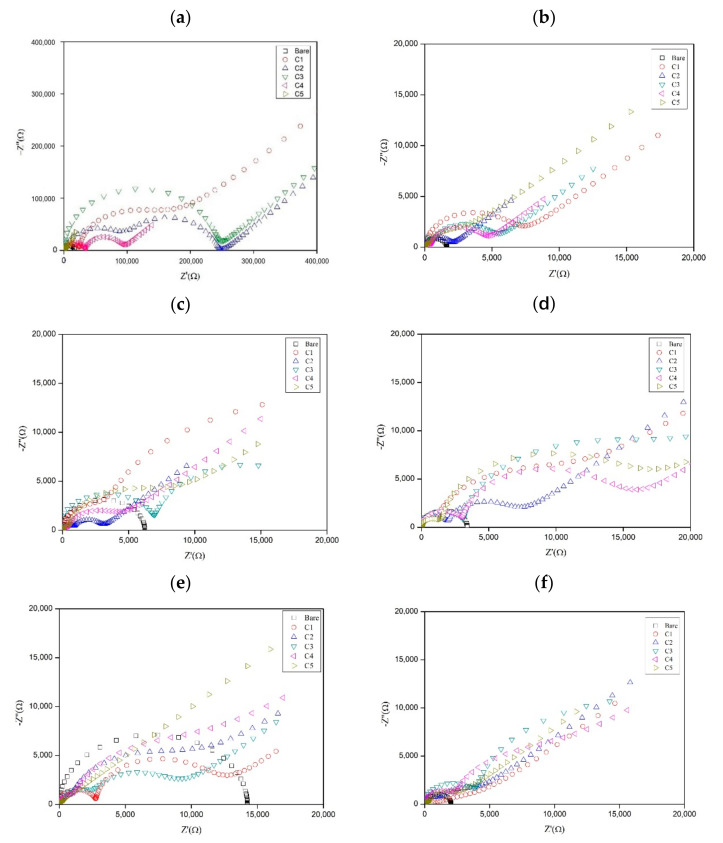
Nyquist plots of the substrate (**a**) before immersion and at (**b**) 10, (**c**) 20, (**d**) 30, (**e**) 40 and (**f**) 50 days of immersion.

The Nyquist plot evaluates the coating performance, corrosion behaviour, and the mechanism imposed by the additives. An equivalent circuit model as showed in Figure 5a consists of *R_Ω_*, *R_ct_*, and *C_dl_*, where *R_Ω_* is the uncompensated resistance between the reference electrode and the test electrode, *R_ct_* is the charge transfer resistance, and *C_dl_* is the double-layer capacitance. In the coating circuit, *R_c_* is the coating resistance and *C_c_* is the coating capacitance. *R_c_* analyzes the degradation of coating performance due to the formation of defects, wherein if the value of *R_c_* increases, the formation of defects decreases. The *C_c_* parameter correlates with the amount of water that enters the coating. Hence, as *C_c_* increases, the amount of water entering also increases. *R_ct_* acts as the indication of the resistance of electron transfer from the metal to the oxidant in the solution, whereas *C_dl_* relates to the delaminated area under the coating. W is the Warburg impedance, a parameter that elucidates the adsorption and mass transport of diffusing species that occur possibly due to the corrosion of the substrate [29]. Figure 5 and Table 3 show the Nyquist plot and data collected for coated and uncoated samples that were immersed for different periods. It is also important to mention that the result for the ‘before immersion’ period was obtained by testing the specimen directly using the test solution collected from the site as soon as the coatings were completely dried. Furthermore, during the collecting process of the immersed specimens, the samples were observed to have multiple patches of solid deposition with various sizes, possibly due to the aggressive formation of the biofilm by bacteria. Although these depositions are believed to have significant impacts during the evaluation of corrosion performance, they were not removed, and the measurements were carried out as is since the study primarily focused on assessing the electrochemical corrosion effects of the real environment towards the fabricated coatings.

According to Table 3, the values of *R_c_* in all the coatings were high; the highest was at C3 (203.00 × 10^3^ Ω). However, as the substrate was immersed, the value of *R_c_* decreased. For the first 10 days of immersion, C3 had the best *R_c_* value, which was 2.44 × 10^3^ Ω, followed by C1 (1.79 × 10^3^ Ω), C2 (1.69 × 10^3^ Ω), C4 (0.70 × 10^3^ Ω), and C5 (0.53 × 10^3^ Ω). A higher *R_c_* value means a stronger resistance of the coating and, subsequently, slower degradation. For 20–50 days of immersion, C3 yielded the greatest *R_c_* value, but its value decreased with time. The amount of water that entered the coating increased as time passed by. This was also observed in other coatings. For the *C_c_* parameter, the best value for 10 days was C1 with 27.90 × 10^−9^ F. As for the other coatings, *C_c_* for C2 was 28.20 × 10^−9^ F, followed by C3 (48.40 × 10^−9^ F), C4 (1170.00 × 10^−9^ F), and C5 (402.00 × 10^−9^ F). As the immersion time increased from 20 days to 40 days, C3 featured the best *R_c_* value, which was lower than for C1, C2, C4, and C5. At 50 days of immersion, the value of *C_c_* increased further.

Next, *R_ct_* and *C_dl_* are parameters related to the phenomenon occuring between the metal and the coating. Overall, for the *R_ct_* value, C3 features the best results in terms of the resistance value as it has the highest value compared to C1, C2, C4, and C5 from 10 to 50 days of immersion. Similarly, in terms of *R_c,_* the trend showed that the resistance decreased when the immersion time increased. For *C_dl_*, at 10 days of immersion, the lowest value was observed in the case of C5, which was 1.18 × 10^−6^ F, followed by C2 (2.38 × 10^−6^ F), C1 (3.35 × 10^−6^ F), C3 (4.32 × 10^−6^ F), and C4 (8.11 × 10^−6^ F). All the values were low compared to the *C_dl_* value of the bare metal, which was 37.60 × 10^−6^ F. For 20 and 30 days of immersion, C3 featured the lowest value of *C_dl_*, but at 40 and 50 days of immersion, the value increased. This was due to the increase in immersion time. The delamination area under the coating increased due to the increase in the water entering the coating, and this corresponded to the *C_c_* value. The interaction of the coating with electrolytes can change the molecular structure of the coating matrix [30]. Overall, C3 with 6 wt% of the KLE containing the major compound of andrographolide featured the best performance according to the performed measurements. At 6 wt%, the coating resistance reached its best value. This limitation was probably due to the coating not being able to absorb the extract and mix it well due to the excessive amount of the KLE [31].

### 2.3. Tafel Polarization

**Figure 6:**
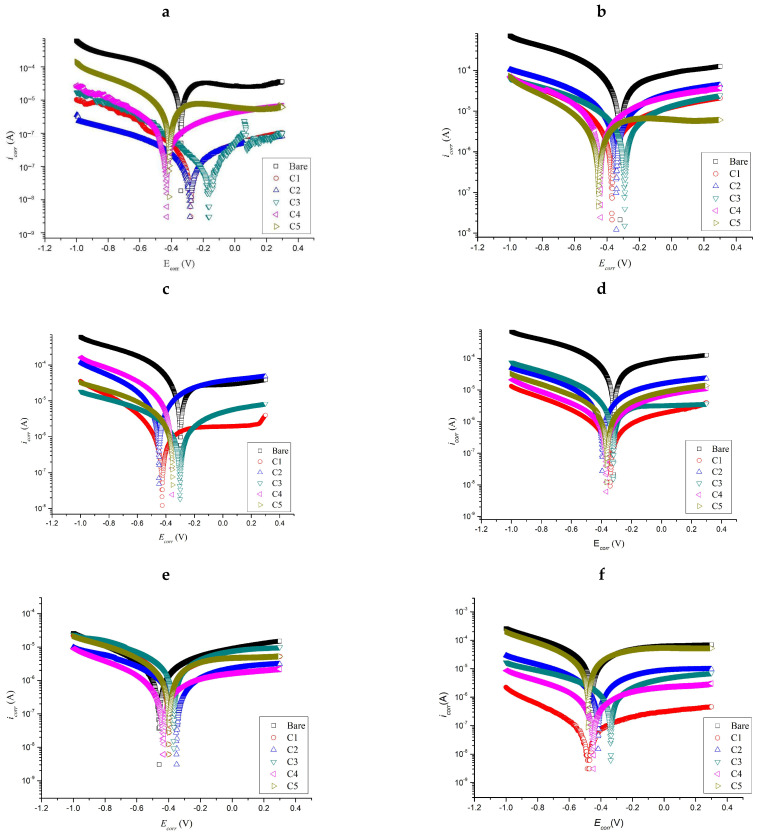
Tafel plots of the substrate (**a**) before immersion and at (**b**) 10, (**c**) 20, (**d**) 30, (**e**) 40, and (**f**) 50 days of immersion.

Figure 6 shows the Tafel plot of coated and uncoated substrates for different immersion times. The study of potentiodynamic polarization analyzes the inhibitive ability of coatings towards corrosion via anodic and cathodic mechanisms. Based on Table 4, the parameters include corrosion current density (i_corr_), corrosion potential (E_corr_), and corrosion rate (CR) (mm/year). According to Table 4, the E_corr_ for bare metal is −0.340 V, whereas i_corr_ is 17.30 × 10^−5^ A/cm^2^. E_corr_ values of substrates with C1, C2, and C3 shifted to the more electropositive region compared to the bare metal, whereas for substrates with C4 and C5, the values shifted towards the more electronegative region. When compared to bare metal, i_corr_ of substrates with a coating is relatively low. These trends show that the coating has a good tendency in retarding the corrosion process because lower i_corr_ values mean good resistance against corrosion. The same applies to the corrosion rate value, showing that a substrate with a coating has a low corrosion value when compared with bare metal. After 10 days of immersion, C3 showed the best performance according to i_corr_ of 3.15 × 10^−5^ A/cm^2^ and CR of 0.05266 mm/year. For the immersion period of 20–50 days, C3 also exhibited the best performance with i_corr_ of 0.16 × 10^−5^ A/cm^2^ and CR of 0.36 mm/year at 20 days. This trend was also observed at 30 days (i_corr_: 0.13 × 10^−5^ A/cm^2^; CR: 21.10 × 10^−4^ mm/year), 40 days (i_corr_: 2.67 × 10^−6^ A/cm^2^; CR: 0.45 × 10^−2^ mm/year) and 50 days (i_corr_: 4.34 × 10^−6^ A/cm^2^; CR: 0.73 × 10^−2^ mm/year) of immersion. Additionally, some out-of-alignment values on the results of C4 were observed during the period of 10−40 days of immersion, where the calculated CR of C4 increased on the 10th and 20th days of immersion but decreased on the following 30th and 40th days. The reason for the initial increase of CR may be due to the degradation of C4, causing a high amount of electrolytes to penetrate the coating and trigger the corrosion reaction to occur faster. As more corrosion product is produced, a passive layer is formed on the exposed area of the coating, which leads to the decrease of corrosion rate observed during the 30th and 40th days of immersion. Other out-of-alignment values were recorded for C3 samples on the 20th day of immersion, possibly due to the damage of the sample during the immersion test. Nevertheless, other samples of C3 throughout the subsequent testing period managed to retain their integrity by displaying good performance. The addition of the best concentration of the KLE additive resulted in a lower i_corr_ value, and this demonstrated that the coating was capable of reducing the corrosion process on the surface of specimens. In addition, C3 that featuerd the best performance in corrosion resistance had a greater tendency to shift E_corr_ to more electropositive values at 10, 40, and 50 days of immersion, but shifted to a more electronegative value at 20 and 40 days. Overall, C3 featured the lowest i_corr_ and CR values among the coatings and bare metal. The incorporation of 6 wt% can be concluded to give the best protection to the metal within the range studies.

### 2.4. Morphology and Element Distribution Studies

**Figure 7:**
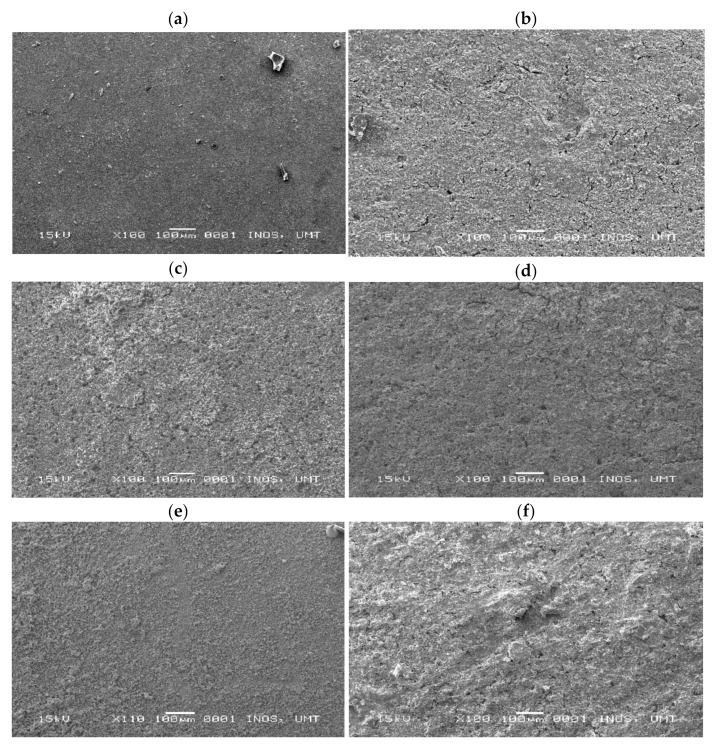
Morphology of (**a**) bare metal, substrates of (**b**) C1, (**c**) C2, (**d**) C3, (**e**) C4, and (**f**) C5 immersed for 50 days.

The surface morphologies of coated and uncoated samples were observed using SEM/EDX to investigate the defect and condition of the coatings after immersion in an aggressive environment for 50 days. Figure 7 shows the samples under 100× magnification. According to Figure 7a, the bare metal showed deposits of unwanted particles such as algae diatoms. The samples that were coated with C1 clearly had cracks and pitting present on their surfaces. The samples of a coating with a 3-wt% additive shown in Figure 7c demonstrated some improvement as compared to the C1 samples. The surface of C2 had fewer cracks and less pitting. According to Figure 7d, the samples that were coated with C3, 6 wt%, had smoother surfaces as compared to C1 and C2. The morphologies in Figure 7e,f showed that there is some inhomogeneity in the paint distribution. The best additive percentage reduced the surface damage, causing the sample to have a smooth surface. Hence, the best performance of a coating was exhibited by C3. However, due to the limitation of the absorption of the KLE, the amount of extract incorporated into the paint matrix affected the overall inhibitor efficiency. The higher percentage of the KLE produced cracks and damage on the surface due to the inhomogeneity of the coating mixture.

Table 5 shows the elemental distribution of sodium (Na), chloride (Cl), chromium (Cr), and nickel (Ni) on the surfaces of bare metal and C3 that were immersed for 50 days. Low chromium concentration was observed under EDX at C3, which was 7.97%, whereas high concentration was observed on bare metal, which was 62.31%. The results supported the findings of electrochemical studies, in which C3 that exhibited the best corrosion control performance was able to reduce the penetration of seawater that might promote formation of chromium oxide, a passive layer commonly formed on SS316L. It is critical to prevent formation of chromium oxide since it is a substance that poses dangerous and toxic effects to aquatic life. Chromium ions have been found to have harmful effects on a few species of fish by affecting their gills, kidney, and liver [32].

### 2.5. Well Diffusion Antimicrobial Test

**Figure 8:**
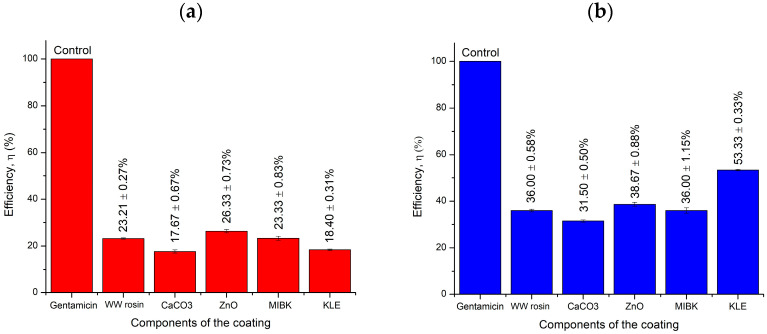
Average inhibition efficiency of each component of the coating against (**a**) *S. aureus* (+) and (**b**) *P. aeruginosa* (−).

**Figure 9:**
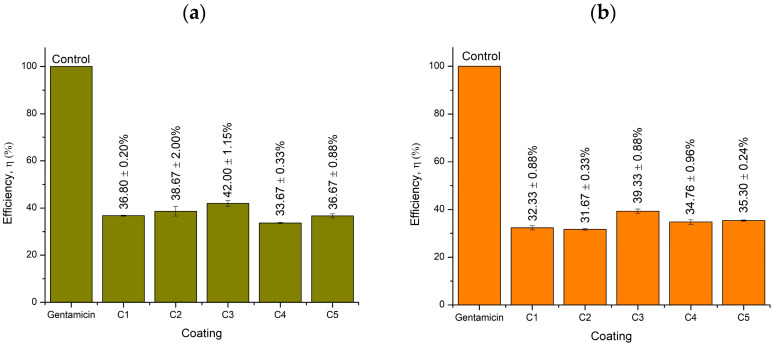
Inhibition efficiency of coatings against (**a**) *S. aureus* (+) and (**b**) *P. aeruginosa* (−).

Figure 8 and Table 6 present the average data collected in experiments on inhibition activities of individual coating components, whereas Figure 9 and Table 7 list the average readings of the inhibition zones of each type of coating. The efficiency was calculated based on the comparison of the samples with antibiotic gentamicin as the positive control. ZnO exhibited high efficiencies of 26% and 39% in inhibiting Gram-positive and Gram-negative bacteria, respectively. Previously, ZnO was investigated and proved to have an excellent potential to inhibit the growth of Gram-positive and Gram-negative bacteria [33]. For the KLE, its inhibition efficiency against gram-positive bacteria (18%) was lower compared with gram-negative bacteria (53%). The crude extract of *A. paniculata* was found to have excellent inhibition properties against gram-positive *S. aureus* (+) and Gram-negative *P. aeruginosa* (−) [34]. Other coating components, namely CaCO_3_, MIBK, and WW rosin, also feature good inhibition activity. The inhibition activity of the combination of these components has been verified, and thus the proposed material has a good potential as an anticorrosive agent in marine environments.

Table 7 presents the average inhibition efficiencies of coatings with different percentages of the KLE against similar bacteria. Only slight differences in inhibition efficiency for both bacterial strains were observed. The highest efficiency against gram-positive bacteria was up to 42% with an inhibition zone of 16 mm for the coating with 6 wt% KLE; it was exhibited by C3. Regarding the Gram-negative bacteria, C3 also featured excellent efficiency in inhibiting bacterial growth at 39% efficiency and 11-mm inhibition zone. The coating as a whole with different wt% of the KLE demonstrated an acceptable performance with regard to inhibition of bacterial growth.

## 3. Materials and Methods

### 3.1. Sample Preparation

Stainless steel 316L bought from CG Tradeware was cut into 25 mm × 25 mm × 3 mm pieces following the American Standard Testing Method (ASTM E3−11) for the preparation of metallographic specimens [35]. The substrate was then polished using Presi Mecapol model P255 U (PRESI, Shah Alam, Malaysia) with several grades of abrasive papers from 240 to 1000 grit, soaked in an acetone solution, rinsed with deionized water, and finally stored in a dry box until use.

### 3.2. Extract Preparation

*A. paniculata* leaves powder purchased from Secret Barn Sdn. Bhd. (Sungai Petani, Malaysia) were soaked in an 85% ethanol solution with a ratio of 1:10. The mixture was placed in a shaker for 24 h, filtered, and then evaporated using a rotary evaporator in a 40 °C water bath until the KLE was obtained. The crude extract was stored in a chiller until use.

### 3.3. Coating Formulation

The coating formulation was developed using WW rosin as a binder, zinc oxide, calcium carbonate as pigments, methyl isobutyl ketone as a solvent, [36] and the *A. paniculata* leaves extract as an additive. The paints were divided into five groups, each having different wt% of the KLE added. The composition of the coating is listed in Table 8. The fabrication of coatings began by mechanically stirring WW rosin with methyl isobutyl ketone (MIBK) at high speed for 4 h. Next, both ZnO and CaCO_3_ were slowly added into the mixture and the stirring was adjusted to 500 rpm. The process was performed for 6 h before the *A. paniculata* leaves extract was added, and the stirring was resumed for another 2 h. The coating thickness of all the samples was standardized at 90 ± 10 µm.

### 3.4. Characterization of the Extract

#### 3.4.1. X-Ray Diffraction (XRD)

XRD is a technique for primary identification of the crystalline state of a material. XRD measurements of the paint and coating samples were conducted using a Rigaku Miniflex II XRD (Rigaku, Shibuya, Japan) to elucidate their properties after the incorporation of the KLE. The analysis began by drying the newly developed coating on a glass surface. The dried coating was then cut into several smaller pieces, where each was placed onto an XRD sample holder. The data obtained from the analysis of these dried specimens was compared with the database in the Search Match software.

#### 3.4.2. Fourier-Transform Infrared Spectroscopy (FTIR)

FTIR was performed using a Thermo Nicolet 380 FTIR spectrometer (Thermo Fisher Scientific, Waltham, MA, USA) to identify the functional group of the KLE. The sample preparation for the study was performed in the same way as described in Section 3.4.1. The extract and the coating samples were analyzed on a KBr disk under infrared ray beams ranging from 4000 to 400 cm^−1^ with the spectrum resolution of 4 cm^−1^. The FTIR data were recorded in the transmittance mode. The transmittance of the infrared rays at different frequencies was translated into an IR absorption plot. The spectral pattern was analyzed and matched according to the IR absorption table to identify the functional groups of the constituents in the extract and the coatings prepared.

#### 3.4.3. High-Performance Liquid Chromatography (HPLC)

The *A. paniculata* extract was diluted with ethanol in a 1:1000 ratio. The extract was then injected into a Shimadzu Prominence HPLC instrument (Shimadzu, Kyoto, Japan) using a Luna 5 µm C18(2) 100 Å column (250 mm × 4.6 mm) with the injection volume of 20 µL, wavelength of 225 nm, and flow rate of 1.0 mL/min [28]. The mobile phases of 20% water (A) and 80% acetonitrile (B) were applied to identify the compound with the highest signal intensity and symmetry. The specimens were measured three times to validate the results.

### 3.5. Immersion Test at the Bay Area of Setiu Terengganu

The substrate was immersed at a site in Merang, Setiu, located at 5°32′17.7″ N, 102°56′44.9″ E. The test was performed to determine the corrosion protection capabilities and mechanical performances of the coating in the real environment. The uncoated and coated substrates with different compositions of the KLE were immersed for 50 days with a batch of specimens taken out every 10 days. The parameters of seawater were measured by a YSI meter, and the result is listed in Table 9.

### 3.6. Electrochemical Studies

#### 3.6.1. Electrochemical Impedance Spectroscopy (EIS)

**Figure 10:**
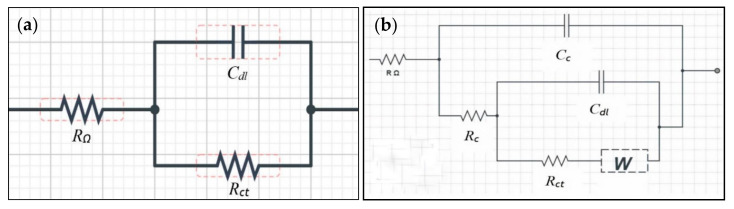
Equivalent circuit for (**a**) bare and (**b**) coated steel.

EIS characterizes the changes of impedance at the surface of a metal due to corrosion. Impedance measurement was performed using Autolab PGSTAT302N (Metrohm, Herisau, Switzerland) controlled by NOVA 10.1 (Metrohm, Herisau, Switzerland) with the frequency ranging between 10 mHz and 100 kHz. It employed simple three-electrode cells, which included the reference electrode (RE) for measuring the potential of the working electrode (WE) as the studied sample and the counter electrode (CE) made of platinum for allowing the current to pass through. The measurement employed the test solution of seawater collected from the testing site and AC signals using peak-to-peak amplitude of 10 mV (rms) for the corrosion potential with the equivalent circuit as displayed in Figure 10 for (a) bare and (b) coated steel [37]. The data obtained were presented in the form of a Nyquist plot.

#### 3.6.2. Potentiodynamic Polarization (PP)

This technique measures the current that flows between the CE and the WE, varied at a selected rate by the application of a potential between the RE and the WE. It is probably the most commonly used polarization testing method for measuring corrosion resistance. The evaluation used a potential ranging from −1.0 V to 0.3 V, referring to the standard electrode of the saturated calomel electrode (SCE), with the scanning rate of 1.0 mV·s^−1^. Information such as anodic charge, passivation potential, and open circuit was obtained from this analysis. The several fixed parameters of stainless steel included were density (8.03 g/cm^3^), equivalent weight (25.65 g/mol), and surface area (6.25 cm^2^).

### 3.7. Morphology Studies

The morphological and elemental distribution of the coated and uncoated stainless steel after 50 days of immersion in the marine environment were investigated using a JSM-6390LA scanning electron microscope with energy-dispersive spectroscopy (SEM-EDS) (JEOL USA, Peabody, MA, USA). The substrates were coated with a fine gold layer using a JFC-1600 Auto Fine Coater with a deposition time of 60 s to obtain a clear image; then they were observed under a SEM. After that, an additional study using EDS was conducted to compare the passive layer formation on bare and coated samples through the identification of elements present on its surface.

### 3.8. Evaluation of Antibacterial Activity

**Figure 11:**
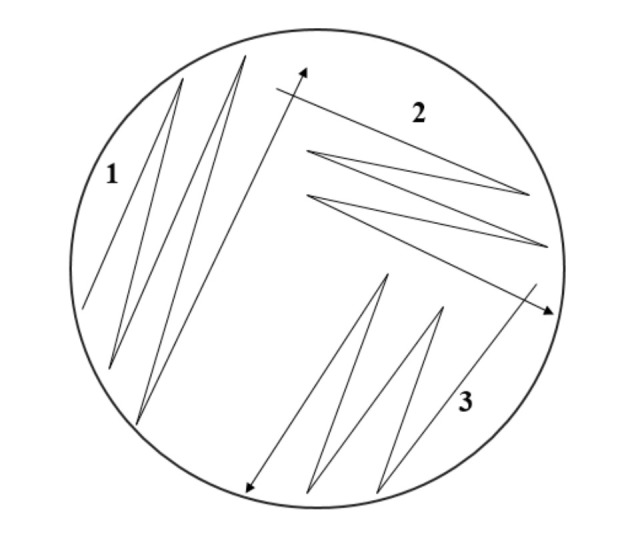
Cross-streaking method for antimicrobial screening.

Muller−Hinton agar (MHA) and nutrient agar (NA) were prepared by dissolving agar powder with distilled water at a certain ratio and autoclaved for 45 min before pouring into a Petri dish. The poured agar was left to solidify before storing in a chiller until use. Nutrient agar (NA) was used to subculture the bacteria. The method used to streak the bacteria is called the cross-streaking method (shown in Figure 11 (reproduced with permission)) [38]. The streaked bacteria were incubated for 18–20 h at 37 °C to allow the bacteria to grow.

After 20 h of incubation, a few colonies from the cultivated bacteria were collected and suspended into a broth and mixed well using a vortex. The turbidity of the sample was measured by a UV−Vis spectrophotometer at the 550-nm wavelength. The bacterial suspension was diluted to obtain a reading of 0.125, equivalent to 0.5 McFarland turbidity [39]. Antibacterial activities of the coatings and of each primary component were evaluated using the well diffusion method. A cork borer with a diameter of 6 mm was used to form wells on the MHA. Two milliliters of each sample were loaded into the wells. Gentamycin was used as the positive control, whereas the C1 coating with 0% KLE was used as the negative control. After 24 h of incubation at 37 °C, the formed zones of inhibition were measured.

## 4. Conclusions

As a conclusion, the use of a plant extract as an additive in the coating yields a good result in reducing the degradation rate of the coating, hence, it also fills in the research gap on the topic of plant application in the field of corrosion in marine applications. According to the FTIR result, the functional groups identified in the KLE such as O−H and R−COOH represent the major component of andrographolide. The notion is further proven by the HPLC analysis where the single compound of andrographolide was observed at a retention time of 4.650 min. The coating with the best concentration of the additive, i.e., 6 wt%, has a good morphological structure based on SEM, EIS, and PP analysis. Such a coating (6 wt%) exhibits the highest value of *R_ct_* (14.00 × 10^3^ Ω) and also the lowest corrosion rate reading (21.10 × 10^−4^ mm/year) when compared to other coatings after 50 days of immersion. The morphology structures of the 6 wt% coating exhibit the least cracks and holes on the surface among another coatings. Additionally, in antimicrobial screening, the C3 coating displayed inhibition zone efficiency up to 42% for *S. aureus* (+) and 39% for *P. aeruginosa* (−). In essence, the addition of the KLE proved to impart a positive impact on the coating performance, but a study focusing on a better incorporation method of the extract into the coating matrix with an evaluation of reproducibility of the results should be further explored to increase the output in future works and allow for a deeper analysis.

## Figures and Tables

**Table 1:** Functional groups and wavenumbers observed in the KLE.

Wavenumber (cm^−1^)	Functional Group	Bond
3348.42	Primary alcohol	O−H
2966.52	Carboxylic acid	R−COOH
1739.79	Amide	C=O
1436.97	Alkane	C−H
1043.49	Ethers	R−O−R

**Table 2:** HPLC analysis data.

Name	Peak	Retention Time (min)	Area (mV*min)	Height (mV)
*A. paniculata* leaves extract	1	4.650	4,589,710	819,072

**Table 3:** Impedance parameters.

Days	Sample	*R_c_* (Ω)	*C_c_* (F)	*R_ct_* (Ω)	*C_dl_* (F)	Warburg Impedance, W (µΩ^−1^)
Before immersion	Bare	*−*	*−*	17.70 × 10^3^	152.00 × 10^−6^	−
	C1	26.40 × 10^3^	12.60 × 10^−9^	124.00 × 10^3^	0.50 × 10^−6^	8.51
	C2	113.00 × 10^3^	1.07 × 10^−9^	129.00 × 10^3^	0.02 × 10^−6^	16.30
	C3	203.00 × 10^3^	159.00 × 10^−9^	202.00 × 10^3^	0.77 × 10^−6^	27.70
	C4	4.75 × 10^3^	208.00 × 10^−9^	29.60 × 10^3^	1.06 × 10^−6^	66.00
	C5	6.32 × 10^3^	37.70 × 10^−9^	38.20 × 10^3^	316.00 × 10^−9^	42.10
10	Bare	−	−	1.65 × 10^3^	37.60 × 10^−6^	−
	C1	1.79 × 10^3^	27.90 × 10^−9^	6.32 × 10^3^	3.35 × 10^−6^	257.00
	C2	1.69 × 10^3^	28.20 × 10^−9^	1.75 × 10^3^	2.38 × 10^−6^	617.00
	C3	2.44 × 10^3^	48.40 × 10^−9^	48.20 × 10^3^	4.32 × 10^−6^	366.00
	C4	0.70 × 10^3^	1170.00 × 10^−9^	3.15 × 10^3^	8.11 × 10^−6^	600.00
	C5	0.53 × 10^3^	402.00 × 10^−9^	2.02 × 10^3^	1.18 × 10^−6^	60.00
20	Bare	−	−	6.23 × 10^3^	79.20 × 10^−6^	−
	C1	5.23 × 10^3^	125.00 × 10^−9^	12.30 × 10^3^	0.58 × 10^−6^	1.33.00
	C2	1.02 × 10^3^	155.00 × 10^−9^	1.82 × 10^3^	2.57 × 10^−6^	403.00
	C3	7.43 × 10^3^	10.70 × 10^−9^	16.30 × 10^3^	0.93 × 10^−6^	103.00
	C4	0.68 × 10^3^	989.00 × 10^−9^	3.15 × 10^3^	4.31 × 10^−6^	198.00
	C5	0.82 × 10^3^	1250.00×10^−9^	6.36 × 10^3^	5.16 × 10^−6^	120.00
30	Bare	−	−	3.40 × 10^3^	85.50 × 10^−6^	−
	C1	2.07 × 10^3^	131.00 × 10^−9^	8.17 × 10^3^	1.55 × 10^−6^	42.00
	C2	2.12 × 10^3^	131.00 × 10^−9^	4.57 × 10^3^	4.20 × 10^−6^	218.00
	C3	3.36 × 10^3^	34.30 × 10^−9^	14.00 × 10^3^	0.51 × 10^−6^	25.00
	C4	3.30 × 10^3^	41.70 × 10^−9^	11.00 × 10^3^	1.34 × 10^−6^	114.00
	C5	1.77 × 10^3^	401.00 × 10^−9^	13.40 × 10^3^	5.08 × 10^−6^	146.00
40	Bare	−	−	14.20 × 10^3^	0.27 × 10^−6^	−
	C1	2.99 × 10^3^	22.90 × 10^−9^	8.58 × 10^3^	2.29 × 10^−6^	171.00
	C2	1.73 × 10^3^	126.00 × 10^−9^	7.57 × 10^3^	0.44 × 10^−6^	29.50
	C3	3.10 × 10^3^	93.40 × 10^−9^	8.22 × 10^3^	107.00 × 10^−6^	95.60
	C4	2.07 × 10^3^	104.00 × 10^−9^	8.09 × 10^3^	0.40 × 10^−6^	18.70
	C5	0.30 × 10^3^	171.00 × 10^−9^	0.68 × 10^3^	0.65 × 10^−6^	31.60
50	Bare	−	−	2.04 × 10^3^	14.60 × 10^−6^	−
	C1	3.89 × 10^3^	2.70 × 10^−9^	2.26 × 10^3^	0.04 × 10^−6^	7.54
	C2	1.46 × 10^3^	209.00 × 10^−9^	2.05 × 10^3^	1.78 × 10^−6^	89.10
	C3	4.37 × 10^3^	275.00 × 10^−9^	15.70 × 10^3^	600.00 × 10^−6^	64.20
	C4	2.78 × 10^3^	43.90 × 10^−9^	7.07 × 10^3^	606.00 × 10^−6^	20.90
	C5	0.34 × 10^3^	1340.00×10^−9^	1.90 × 10^3^	10.50 × 10^−6^	294.00

**Table 4:** Polarization parameters.

Days	Sample	E_corr_ (V)	i_corr_ (A/cm^2^)	CR (mm/year)
Before immersion	Bare	−0.340	17.30 × 10^−5^	28.99 × 10^−2^
	C1	−0.279	0.01 × 10^−5^	2.10 × 10^−4^
	C2	−0.282	0.08 × 10^−5^	13.10 × 10^−4^
	C3	−0.163	0.04 × 10^−6^	0.70 × 10^−4^
	C4	−0.430	0.32 × 10^−5^	5.40 × 10^−4^
	C5	−0.415	2.98 × 10^−5^	4.99 × 10^−2^
10	Bare	−0.318	4.20 × 10^−5^	78.88 × 10^−2^
	C1	−0.396	5.20 × 10^−5^	8.69 × 10^−2^
	C2	−0.342	5.53 × 10^−5^	9.23 × 10^−2^
	C3	−0.290	3.15 × 10^−5^	5.27 × 10^−2^
	C4	−0.438	5.60 × 10^−5^	9.36 × 10^−2^
	C5	−0.454	6.17 × 10^−5^	10.32 × 10^−2^
20	Bare	−0.298	19.10 × 10^−5^	0.10 × 10^−2^
	C1	−0.426	0.62 × 10^−5^	56.93 × 10^−2^
	C2	−0.449	3.26 × 10^−5^	1.02
	C3	−0.301	0.16 × 10^−5^	0.36
	C4	−0.356	7.44 × 10^−5^	1.12
	C5	−0.352	0.69 × 10^−5^	0.75
30	Bare	−0.310	47.00 × 10^−5^	0.79
	C1	−0.374	0.49 × 10^−5^	5.70 × 10^−2^
	C2	−0.394	4.64 × 10^−5^	7.75 × 10^−2^
	C3	−0.321	0.13 × 10^−5^	21.10 × 10^−4^
	C4	−0.367	0.66 × 10^−5^	1.10 × 10^−2^
	C5	−0.366	2.29 × 10^−5^	3.80 × 10^−2^
40	Bare	−0.459	4.55 × 10^−6^	0.76
	C1	−0.405	3.44 × 10^−6^	0.64 × 10^−2^
	C2	−0.349	1.20 × 10^−6^	1.40 × 10^−2^
	C3	−0.368	2.67 × 10^−6^	0.45 × 10^−2^
	C4	−0.432	4.39 × 10^−6^	0.73 × 10^−2^
	C5	−0.483	8.46 × 10^−6^	0.14
50	Bare	−0.457	18.80 × 10^−5^	0.31
	C1	−0.483	0.18 × 10^−6^	1.39 × 10^−2^
	C2	−0.417	2.04 × 10^−5^	3.41 × 10^−2^
	C3	−0.337	4.34 × 10^−6^	0.73 × 10^−2^
	C4	−0.446	1.10 × 10^−5^	1.84 × 10^−2^
	C5	−0.482	8.88 × 10^−5^	0.15

**Table 5:** Elemental composition of the metals immersed for 50 days as measured by EDX.

Element	Mass (%)	
	Bare	C3 (6%)
Na	0.84	43.63
Cl	0.47	38.69
Cr	62.31	9.71
Ni	36.39	7.97
Total	100.00	100.00

**Table 6:** Inhibition properties of each coating component.

Sample	*S. Aureus* (+)			*P. Aeruginosa* (−)		
	Average Inhibition Zone (mm)	Efficiency (%)	Standard Deviation	Average Inhibition Zone (mm)	Efficiency (%)	Standard Deviation
Antibiotics	38	−	0	28	−	0
ZnO	10	26	1.26	11	39	1.53
CaCO_3_	7	18	1.15	9	32	0.87
MIBK	9	23	1.44	10	36	2.00
WW rosin	9	23	0.47	10	36	1.00
KLE	7	18	0.53	15	53	0.33

**Table 7:** Inhibition properties of coatings with different KLE concentration.

Sample	*S. Aureus* (+)			*P. Aeruginosa* (−)		
	Inhibition Zone (mm)	Efficiency (%)	Standard Deviation	Inhibition Zone (mm)	Efficiency (%)	Standard Deviation
Antibiotics	38	−	0	28	−	0
C1 (0 wt%)	14	37	0.35	9	32	1.53
C2 (3 wt%)	15	39	3.50	9	32	0.58
C3 (6 wt%)	16	42	2.00	11	39	1.53
C4 (9 wt%)	13	34	0.58	10	35	1.66
C5 (12 wt%)	14	37	1.53	10	35	0.42

**Table 8:** Composition of the coatings in weight percentage (wt%).

Component/Coating	C1	C2	C3	C4	C5
WW rosin	49.1	49.1	49.1	49.1	49.1
MIBK	20.0	20.0	20.0	20.0	20.0
CaCO_3_	10.9	10.9	10.9	10.9	10.9
ZnO	20.0	17.0	14.0	11.0	8.0
KLE	0.0	3.0	6.0	9.0	12.0

**Table 9:** Parameters of seawater.

Parameter	Average Reading	Standard Deviation
Dissolved oxygen, DO (mg/L)	5.06	0.23
Conductivity (mS)	43.6	0.31
Salinity (ppt)	26.4	0.25
pH	8.1	0.22

## Data Availability

The data presented in this study are available upon request from the corresponding author.

## Sample Availability

Samples of the compounds not are available from the authors.
